# Emergence of Multiple Polaritonic States of Water‐in‐Salt in Cavity

**DOI:** 10.1002/nap2.70256

**Published:** 2026-08-17

**Authors:** Tomohiro Fukushima, Keiji Moriya, Soushi Yoshimitsu, Masaki Itatani, Kei Murakoshi

**Affiliations:** ^1^ Department of Chemistry, Faculty of Science Hokkaido University Sapporo Japan; ^2^ Precursory Research for Embryonic Science and Technology (PRESTO), Japan Science and Technology Agency (JST) Tokyo Japan

**Keywords:** cavity, electrolyte, polariton, water

## Abstract

Water‐in‐salt electrolytes exhibit multiple spectroscopically isolated OH stretching modes owing to spatially and temporally isolated water species. Here we show that these modes couple cooperatively to a Fabry–Perot cavity, yielding well‐resolved collective polaritonic states. These characteristics provide an insight to distinguishing the light–matter modes of water which have remained unresolved due to the common assumption of homogeneous broadening. This demonstrates that vibrational polariton formation in aqueous media is dictated by the vibrational heterogeneity that remains spectroscopically distinguishable whenever structural exchange occurs more slowly than the light–matter coupling timescale, establishing water‐in‐salt electrolytes as an ideal platform for disentangling multi‐mode light–matter interactions in molecular liquids.

## Graphical abstract

Water‐in‐salt electrolytes exhibit multiple hydration‐specific OH stretching modes that cooperatively couple to a Fabry–Perot cavity, generating distinct polaritonic states. Unlike conventional water systems described by approximated homogeneous broadening, the observed multimode polariton formation reflects the interplay between vibrational heterogeneity and slow structural exchange dynamics in concentrated aqueous electrolytes.

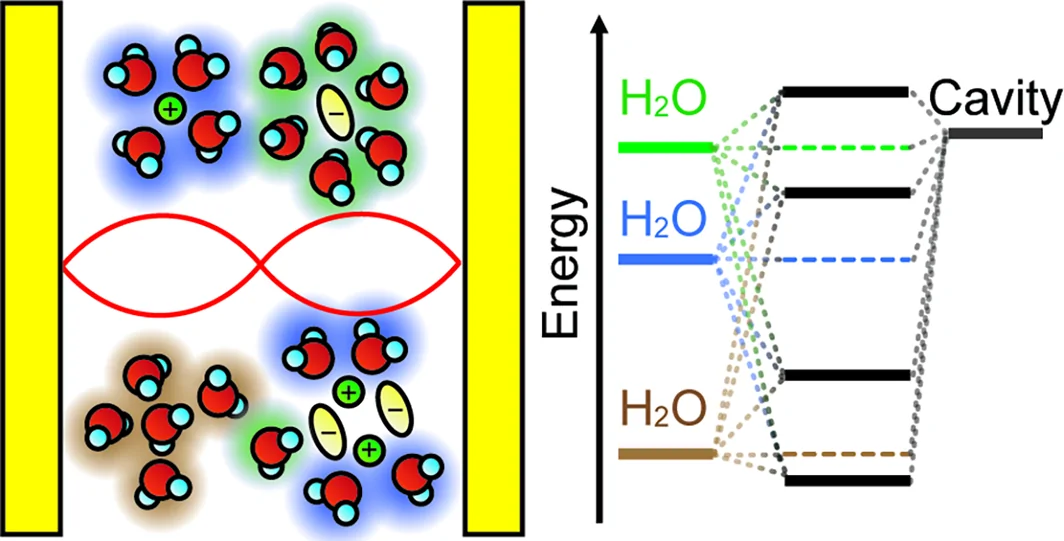

## 1 Introduction

Recently, there are emerging interests in the strong coupling of molecular transition to the vacuum electromagnetic field that unlocks unique possibilities in chemistry [1, 2, 3, 4, 5, 6, 7, 8]. In particular, vibrational strong coupling (VSC) of water has recently been shown to modulate diverse physicochemical processes [9, 10, 11, 12, 13, 14, 15, 16, 17, 18, 19, 20, 21, 22, 23, 24], yet, the fundamental nature of the resulting polaritonic states remains unresolved [14, 18, 21]. Although liquid water contains a distribution of hydrogen‐bonding environments [25, 26, 27, 28, 29], previous experimental studies on VSC have consistently reported the approximated polaritonic states as a two‐level system. This apparent simplicity has been originated from the OH stretching behaviour assuming a homogeneously broadened oscillator [30]. However, the extent to which hydrogen‐bond exchange obscures the intrinsic vibrational heterogeneity remains unclear. Distinct solvation environments generate multiple vibrational modes, which can in turn give rise to multiple polaritonic states under cavity coupling [31].

Here, we demonstrate that water‐in‐salt electrolytes exhibit multiple polaritonic states arising from distinct hydration‐specific vibrational modes. By directly comparing bulk water and highly concentrated solutions inside a Fabry–Perot cavity, we observed polariton formation to multiple Rabi splitting originating from discrete OH stretching species. The polaritonic behaviour itself is expected to reflect the underlying molecular coupling: homogeneous broadening should yield a pseudo‐two‐level polariton formation, whereas inhomogeneous broadening arising from spectroscopically distinct vibrational sub‐ensembles should give rise to a multi‐level polaritonic formation. This study provides new insight into how molecular heterogeneity governs polariton formation in aqueous systems.

## 2 Materials and Methods

Lithium bis(trifluoromethanesulfonyl)imide, denoted as LiTFSI was purchased from Tokyo Chemical Industry. LiTFSI aqueous solution was prepared according to the literature procedure [32]. Au was purchased from Tanaka Precious Metals, Japan. The CaF_2_ windows were purchased from Specac Ltd., UK. All aqueous electrolyte solutions were prepared using Milli‐Q water (Millipore; 18.2 MΩ cm resistivity).

Spectroscopic measurements were conducted based on the established procedure [18, 19, 20]. Infrared (IR) spectroscopic measurements were recorded using a JASCO FT‐IR 4200. The resolution was set as 4 cm^−1^, and the spot size was typically 2.5 mm in diameter. The quality of the cavity cell was confirmed based on the cavity mode observation in IR spectra. The quality of the cavity cell was also confirmed from the interference colour fringe by eye. Spectroscopic fitting was conducted by using pseudo‐Voigt functions.

## 3 Results and Discussion

We utilised LiTFSI as an electrolyte that can reach the solubility of 21 mol L^−1^. The structural environments in concentrated electrolytes can be classified into contact ion pairs, aggregated ion clusters and free water molecules [33, 34, 35]. Figure 1a shows the IR spectra of LiTFSI aqueous solution with the concentration of 21 mol L^−1^. Compared with the broad IR spectra of water (Supporting Information S1: Figure S1), the multiple narrow bands were observed. The vibrational mode of water molecules in LiTFSI aqueous solution can be assigned to the different vibrational modes based on the hydration structure [25]. The vibrational mode of bulk‐like water can be observed at 3340 cm^−1^. The vibrational modes of ion‐bound water were observed at 3580 cm^−1^ (Li^+^) and at 3700 cm^−1^ (TFSI^−^) [34, 36, 37]. As shown in Supporting Information S1: Figure S1, the concentration of LiTFSI electrolytes in water is critical for the vibrational structure of electrolyte solution. For example, the sharp OH stretching mode was broadened upon the decrease of the ionic strength. The observed behaviour is unique for the less hydrogen‐bonding systems in water‐in‐salt electrolyte solutions.

**FIGURE 1:**
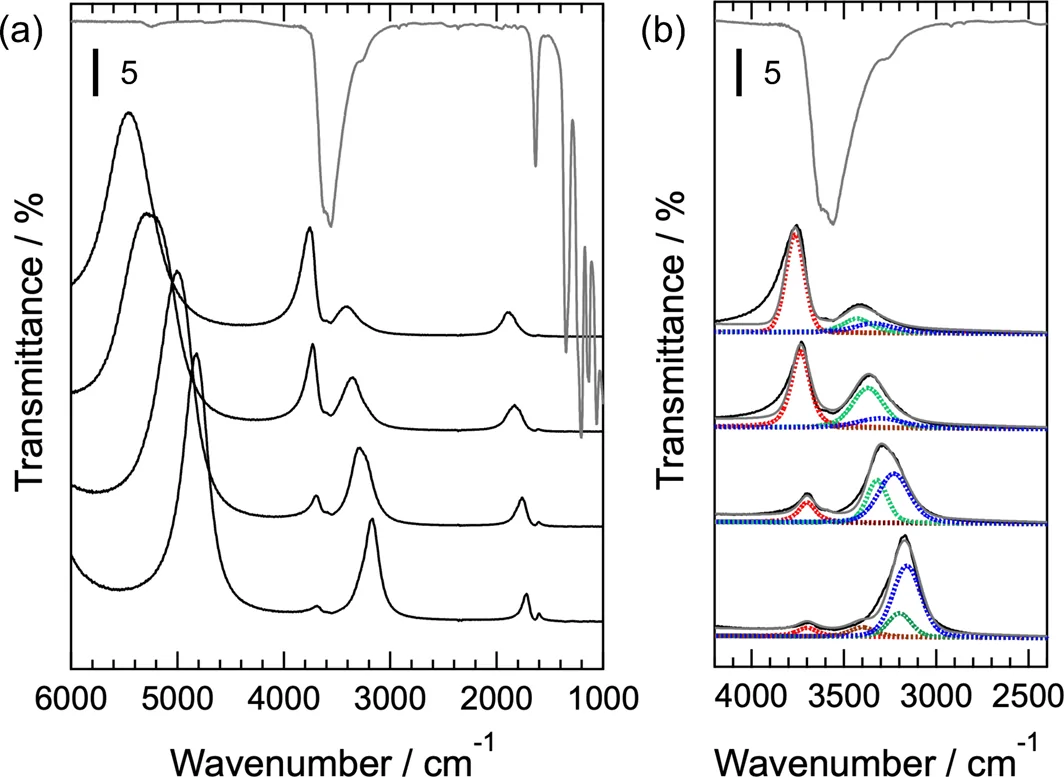
(a) IR spectra of the LiTFSI solution (21 mol L^−1^) in a non‐cavity cell (grey) and LiTFSI solution (21 mol L^−1^) in a cavity cell (black). From top to bottom, cavity length was adjusted to the 2.0, 2.1, 2.2, and 2.3 μm. From top to bottom, the second order of cavity mode frequency corresponded to 3630, 3490, 3330, or 3210 cm^−1^. (b) The peak assignment of polaritonic behaviour of OH stretching mode of LiTFSI (21 mol L^−1^) in a cavity. From the higher energy polaritonic states, the pseudo‐Voigt fitting lines were drawn as red, brown, green, and blue lines. The sum of the pseudo‐Voigt fitting lines was drawn in grey.

The cavity was prepared according to the previous literature [18, 19, 20]. The mirrors were fabricated by depositing 20‐nm‐thick SiO_2_, 8‐nm‐thick Au and 2‐nm‐thick Ti on a 2‐mm‐thick CaF_2_ flat window. Approximately 50 μL of LiTFSI 2.5 H_2_O was dropped on the mirror. The mirrors were sandwiched to create a home‐made cell, and the cavity length (*L*) was tuned using a screw as same method as previously report [18, 19, 20]. The cavity modes were determined using the following equations: *ω*_cav_(*i*) = *ik*_0_ and *k*_0_ = (2 *nL*)^−1^, where *ω*_cav_(*i*) is the frequency of the *i*th cavity mode (cm^−1^), *i* is an integer, *k*_0_ is the free spectral range corresponding to the peak‐to‐peak separation of cavity modes (cm^−1^), *n* is the refractive index of the cavity medium and *L* is the cavity length (μm). *L* was estimated by considering the refractive index of LiTFSI solution as 1.382 [38].

The IR spectra of the LiTFSI solution in the cavity were examined as shown in Figure 1a. Peak positions of cavity modes were dependent on the cavity length. When the *L* was close to the 2.0–2.3 μm, the second order of the cavity mode was coupled to the OH stretching mode of LiTFSI solution. Because of the presence of multiple OH stretching modes in LiTFSI solution, the multiple peaks were observed. Pseudo‐Voigt functions were employed because the observed cavity–polariton transmission peaks contain both Lorentzian and Gaussian components. The Lorentzian component phenomenologically represents the coherent cavity or photon‐like contribution, whereas the Gaussian component accounts for the distribution of local hydrogen‐bonding environments that produces inhomogeneous broadening of the OH stretching vibrations in concentrated water‐in‐salt electrolytes. The OH stretching bands in concentrated electrolyte solutions are dominated by distributions of local hydrogen‐bonding environments, which are commonly represented as inhomogeneously broadened spectral features [25, 39]. As a result, four pseudo‐Voigt peaks were observed (Figure 1b and Supporting Information S1: Figure S2), consistent with the coupling of three discrete vibrational oscillators with a single cavity mode. The polaritonic behaviours are sensitive to the cavity modes; the peak position and linewidth are dependent on the cavity modes. The observed asymmetric peak shapes can originate from complex light–matter interactions under multimode coupling conditions [22, 31, 40, 41, 42]. The sharper and more distinguishable OH stretching mode of the water‐in‐salt electrolyte in cavity suggest reduced configurational averaging compared with bulk water in cavity [14, 18]. Such suppression of rapid spectral diffusion enables individual hydration‐specific vibrational modes to persist on timescales relevant to cavity coupling, thereby facilitating the emergence of multiple polaritonic branches. In addition, the broad overlap observed outside the cavity contrasts with the more distinguishable polaritonic features observed under cavity coupling in highly concentrated LiTFSI solutions.

In such a multi‐oscillator strong‐coupling regime, *N* vibrational modes hybridise with one cavity photon to produce *N* + 1 polaritonic states—matching the four mixed states observed here. The observed behaviour resembles previously reported multiple Rabi‐splitting phenomena arising from co‐resonators coupled to a single cavity mode [43, 44] or coupling of the single oscillator modes with the multiple cavity modes [11, 31, 45]. The dispersion behaviour of vibrational polariton was examined by plotting peak positions of four polaritonic states and corresponding cavity modes (Figure 2). Anti‐crossing behaviour was observed for polaritonic peaks composed of light line and OH stretching vibrational modes of LiTFSI solution. The estimated Rabi splitting energy at resonance is 100 cm^−1^ (bulk‐like water), 150 cm^−1^ (Li^+^‐bound water) and 150 cm^−1^ (TFSI^−^‐bound water).

**FIGURE 2:**
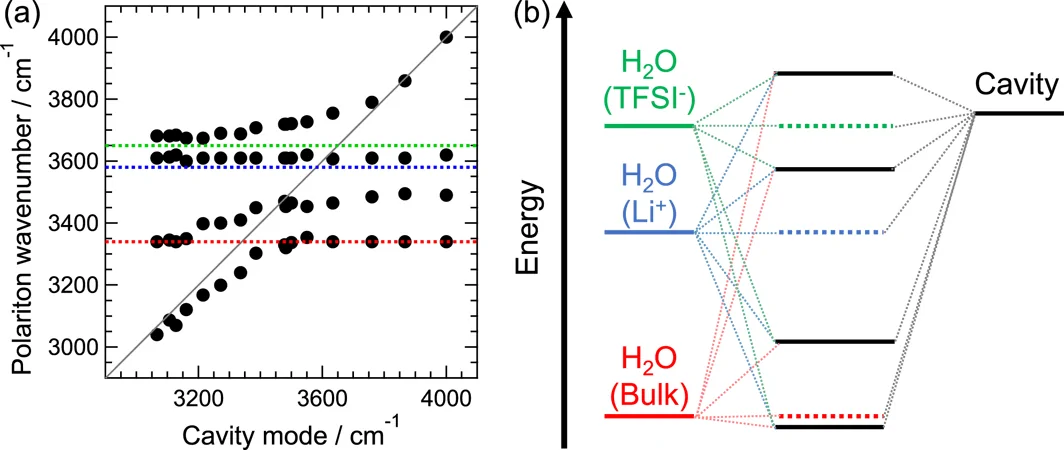
(a) Relationship between peak dispersion and cavity mode. Grey line: light line, red dotted line 3340 cm^−1^ OH stretching mode of bulk‐like water. Blue dotted lines: 3580 cm^−1^ OH stretching mode of Li^+^‐bound water. Green dotted line: 3700 cm^−1^ OH stretching mode of TFSI^−^‐bound water. (b) Energy diagram of the multiple‐polaritonic state formation from second order of cavity mode and OH stretching mode from bulk, Li^+^ and TFSI^−^‐bound H_2_O. Dark states are drawn as dotted lines.

To examine the vibrational polariton behaviour, the concentration‐dependent Rabi splitting behaviour was examined. Supporting Information S1: Figures S3 and S4 showed the concentration‐dependent IR spectra and dispersion behaviour of the LiTFSI aqueous solution in the cavity. The polaritonic behaviour is different from the highly concentrated LiTFSI solution. In case with the water molecules, the polaritonic behaviours have been described with the single peaks in the upper polaritons and lower polaritons [9, 10, 11, 18, 19, 20]. Even in the presence of multiple different hydrogen bonding mode, those peaks were assumed to be homogeneously broadened under the vibrational polariton formation to form the single pair of upper and lower polaritons [14, 30].

The overall reduction in the Rabi splitting with decreasing water concentration is qualitatively consistent with the expected $\sqrt{N}$ dependence of collective vibrational coupling. However, unlike an ideal single‐oscillator system, the present electrolyte contains multiple hydration‐dependent vibrational sub‐ensembles, including bulk‐like water, Li^+^‐bound water and TFSI^−^‐bound water, each contributing differently to the effective oscillator strength. In particular, the lower polaritonic branch is strongly influenced by the bulk‐like water component, whose relative population changes substantially with electrolyte concentration. In contrast, the upper polaritonic features associated with hydration specific OH stretching modes remain at relatively similar frequencies, resulting in a comparatively weak apparent concentration dependence of the upper branch.

The experimental linewidths are systematically smaller than the Hopfield‐coefficient‐weighted reference linewidths (see Supporting Information S1: Figures S5 and S6), indicating qualitative linewidth narrowing. This weighted‐average comparison follows the same logic used to first establish motional narrowing of cavity polaritons in semiconductor microcavities, where the observed linewidth falls below the value expected from a simple Hopfield‐weighted average of the exciton and photon linewidths [46, 47, 48]. The qualitative narrowing trend we observe is also consistent with recent reports of motional narrowing in molecular cavity–polariton systems, where suppression of excitonic disorder was similarly attributed to photonic exchange between the delocalised cavity mode and the disordered molecular ensemble rather than to a fully quantitative linewidth model [49, 50]. This description is further supported by recent theoretical treatments that derive Hopfield‐coefficient‐weighted linewidth expressions for the lower and upper polariton branches and predict subaverage narrowing consistent with the observed trend [51]. The reduced linewidth is consistent with suppression of disorder‐induced broadening through photonic delocalisation. Although the present analysis is not intended as a quantitative motional‐narrowing model, the observed trend supports that the hybrid states extend over multiple hydration sub‐ensembles rather than behaving as uncoupled vibrational resonances.

Highly concentrated water‐in‐salt electrolytes such as LiTFSI disrupt the continuous hydrogen‐bonding landscape and generate several well‐defined hydration species. Bulk‐like water, Li^+^‐bound water and TFSI^−^‐bound water each possess distinct vibrational signatures reflecting their different local coordination environments. Importantly, the exchange time scale among these species is around 10–100 ps [28, 36, 52, 53, 54, 55] whereas hydrogen‐bond rearrangement occurs within 1 ps in water [56, 57]. Because the interconversion among hydration species is slower than the vibrational dephasing time, each OH mode persists long enough to couple to the cavity field. When placed in a Fabry–Perot cavity, these discrete vibrational modes couple cooperatively to the cavity field, giving rise to extended polaritonic states. This behaviour is consistent with a clear separation of timescales: The sub‐200 fs Rabi period associated with the observed splitting is much shorter than the picosecond‐scale structural exchange among hydration species, so the distinct sub‐ensembles appear quasi‐static on the timescale relevant to polariton formation, analogous to the vibronic decoupling mechanism [58]. It is this separation of timescales, rather than a direct dynamical influence of structural exchange on polariton formation, that allows vibrational heterogeneity to manifest as distinct polaritonic branches. We emphasise that the multi‐level polaritonic manifold observed here is not simply a spectroscopic readout of coexisting hydration species already discernible in linear absorption. Rather, it reflects a transition from the homogeneously broadened pseudo‐two‐level coupling regime characteristic of ordinary water to an inhomogeneously broadened, multi‐level coupling regime enabled by suppressed structural exchange in water‐in‐salt electrolytes. These insights establish that polariton formation in aqueous media is governed by vibrational inhomogeneity, which shows distinct polaritonic branches as long as structural exchange among hydration species remains slower than the light–matter coupling timescale, providing a mechanistic framework for understanding cavity responses in complex liquid systems. They also demonstrate that vibrational polaritons cannot be treated within an idealised two‐level picture. Instead, multiple vibrational modes, together with possible contributions from overtones, Fermi resonances or weak mode mixing, must be considered to accurately describe the formation of polaritonic states. Incorporating these multimode effects is therefore essential for a comprehensive understanding of polariton behaviour in molecular liquids.

## 4 Conclusion

In conclusion, we demonstrate that water‐in‐salt electrolytes possess distinct hydration structures that generate multiple polaritonic states. The emergence of multiple polaritonic states stands in sharp contrast to the homogeneous broadening that governs VSC in water. These findings reveal that polariton formation in liquid water systems is governed by vibrational heterogeneity, whose distinct sub‐ensembles remain resolvable because structural exchange proceeds on a much slower timescale than the light–matter coupling. This study provides a mechanistic foundation for controlling polaritonic behaviour in liquid‐phase systems and highlight the importance of individual vibrational modes in determining polaritonic responses in complex liquid.

## Author Contributions

**Tomohiro Fukushima:** conceptualization, investigation, funding acquisition, writing – original draft, writing – review and editing, visualization, formal analysis, supervision, data curation, resources, project administration, methodology, validation. **Keiji Moriya:** investigation, writing – review and editing, formal analysis, data curation. **Soushi Yoshimitsu:** data curation, formal analysis, writing – review and editing. **Masaki Itatani:** writing – review and editing, writing – original draft. **Kei Murakoshi:** writing – original draft, writing – review and editing, funding acquisition, conceptualization, project administration, resources, supervision.

## Funding

This work was supported by JSPS KAKENHI (JP20H05281, JP21K14596, JP22H02023, JP22K18315, JP25H00862, JP25K22193, and JP26K01452). Support by the JST‐Mirai Programme (JPMJMI21EB), GteX Programme (JPMJGX23H2), the JST‐PRESTO programme: Exploring Quantum Frontiers Through Quantum‐Classical Interdisciplinary Fusion (JPMJPR23FB), the Photo‐excitonix Project at Hokkaido University, and the MEXT Programme: Data Creation and Utilisation‐Type Material Research and Development Project (JPMXP1122712807) is also acknowledged.

## Conflicts of Interest

The authors declare no conflicts of interest.

## Supporting information


Supporting Information S1

## Data Availability

The datasets generated and/or analysed during the current study are available from the corresponding author upon reasonable request.
