# Supplementary material for: Emergence of Multiple Polaritonic States of Water‐in‐Salt in Cavity
Source: Nanophotonics. 2026 Aug 17;15(16):e70256. doi: 10.1002/nap2.70256 (PMC13672149; doi:10.1002/nap2.70256)
Supplement: Supplementary file 1 [file NAP2-15-e70256-s001.pdf]

Supporting Information

Emergence of Multiple Polaritonic States of Water-in-Salt in Cavity

Tomohiro Fukushima, Keiji Moriya, Soushi Yoshimitsu, Masaki Itatani, Kei Murakoshi

Department of Chemistry, Graduate School of Science, Hokkaido University,

Sapporo, Hokkaido, 060-0810, Japan

Precursory Research for Embryonic Science and Technology (PRESTO), Japan Science and Technology Agency (JST), 4-1-8 Honcho, Kawaguchi, Saitama 332-0012, Japan.

Line-width analysis of the multiple polaritonic branches

To analyze the linewidths of the multiple polaritonic branches, the experimental dispersion was first fitted using a following Hamiltonian consisting of one cavity photon mode and three OH stretching vibrational modes corresponding to bulk-like water, Li^+-bound water, and $TFSI^-$-associated water:

$$H = \begin{pmatrix} E_{\text{cav}} & g_1 & g_2 & g_3 \\ g_1 & E_{\text{TFSI}} & 0 & 0 \\ g_2 & 0 & E_{\text{Li}} & 0 \\ g_3 & 0 & 0 & E_{\text{bulk}} \end{pmatrix}$$

The eigenvalues of this Hamiltonian reproduce the measured polariton dispersion, while the corresponding eigenvectors provide the Hopfield coefficients $|C_i|^2$, which represent the photonic and vibrational fractions of each polariton branch. Obtained polariton branches were noted as P1, P2, P3, and P4 from higher to lower energy.

Using these Hopfield coefficients, the reference linewidth of each polariton branch was calculated as following

$$\Gamma_{\text{calc}} = \sum |C_i|^2 \Gamma_i$$

Γ_i are the linewidths of the cavity and three uncoupled OH stretching bands obtained from pseudo-Voigt fitting of the non-cavity IR spectrum. The calculated linewidths were then compared with the experimentally determined polariton linewidths extracted from pseudo-Voigt fitting of the cavity transmission spectra as shown in Figure S6.

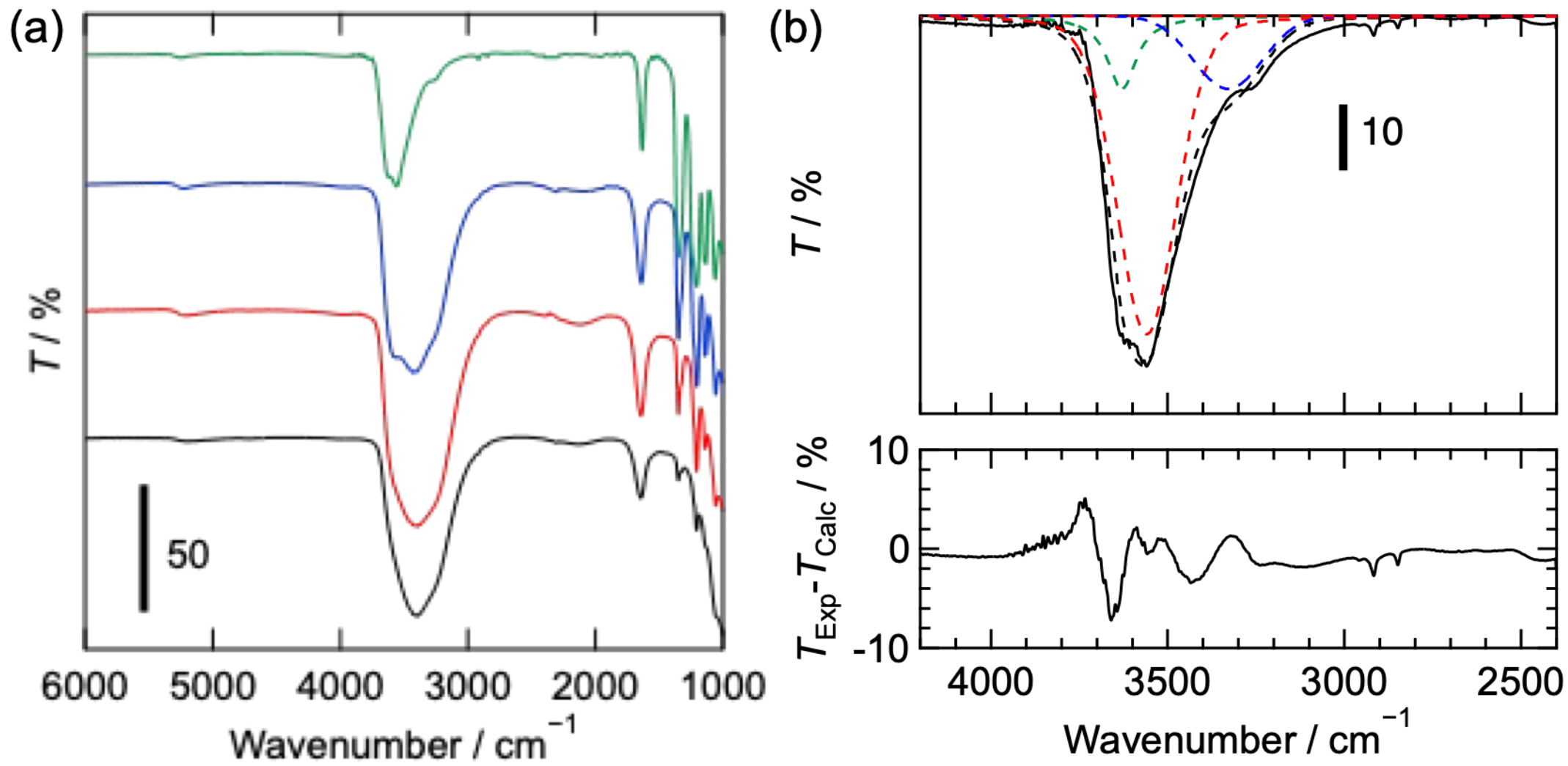

Fig. S1. (a) IR spectroscopy of the LiTFSI electrolyte solution with the variable concentration. Concentration of LiTFSI electrolyte is 21 mol L^{-1} (green), 10.5 mol L^{-1} (blue), 4.2 mol L^{-1} (red), 2.1 mol L^{-1} (black). (b) Representative decomposition of the OH components of LiTFSI electrolyte (21 mol L^{-1}) with pseudo-Voigt function. Residual spectrum between the experimental and fitted spectra were shown in the bottom.

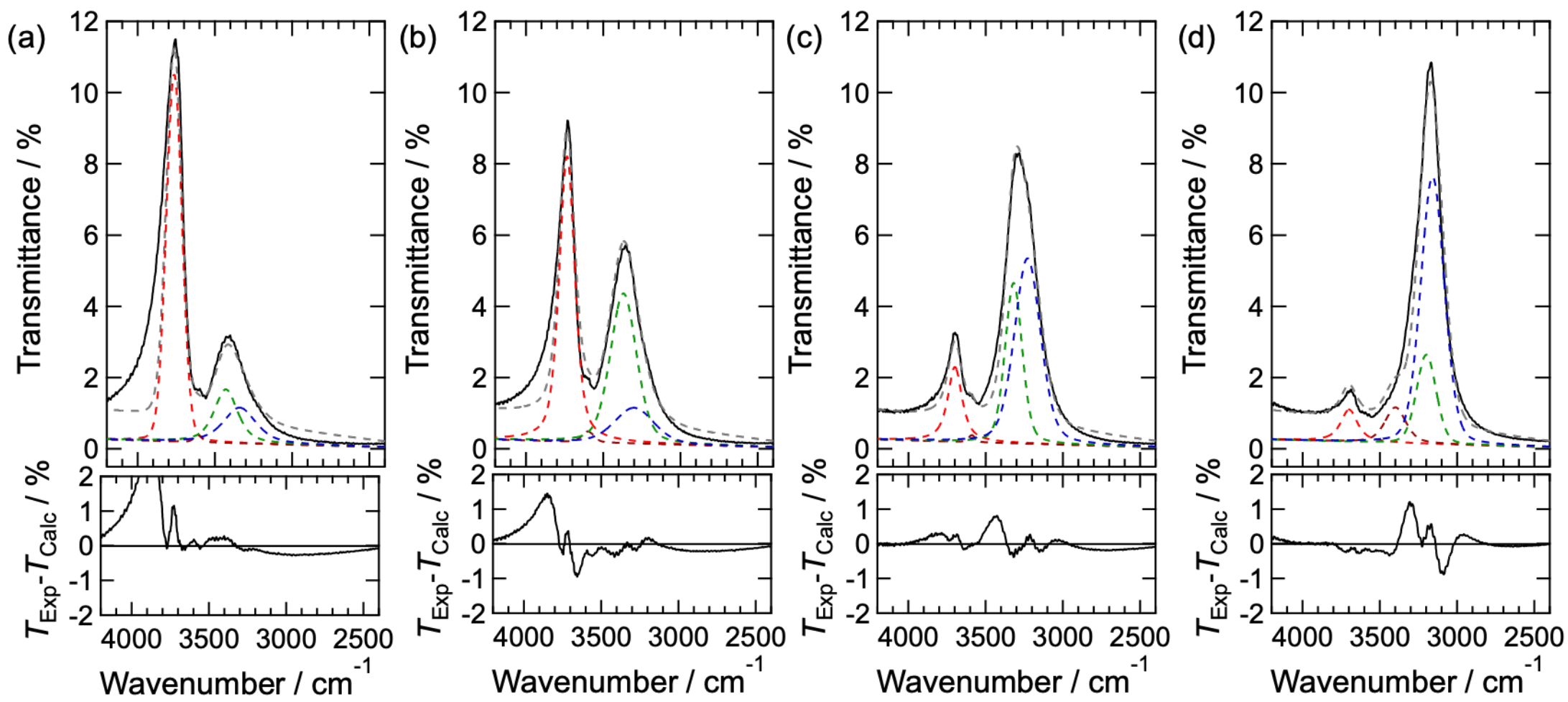

Fig. S2. Representative fitting of the IR transmission spectra of LiTFSI solution (21 mol L^{-1}) in a cavity cell (black) with the fitting components of the pseudo-Voigt fitting. Residual spectrum between the experimental and fitted spectra were shown in the bottom.

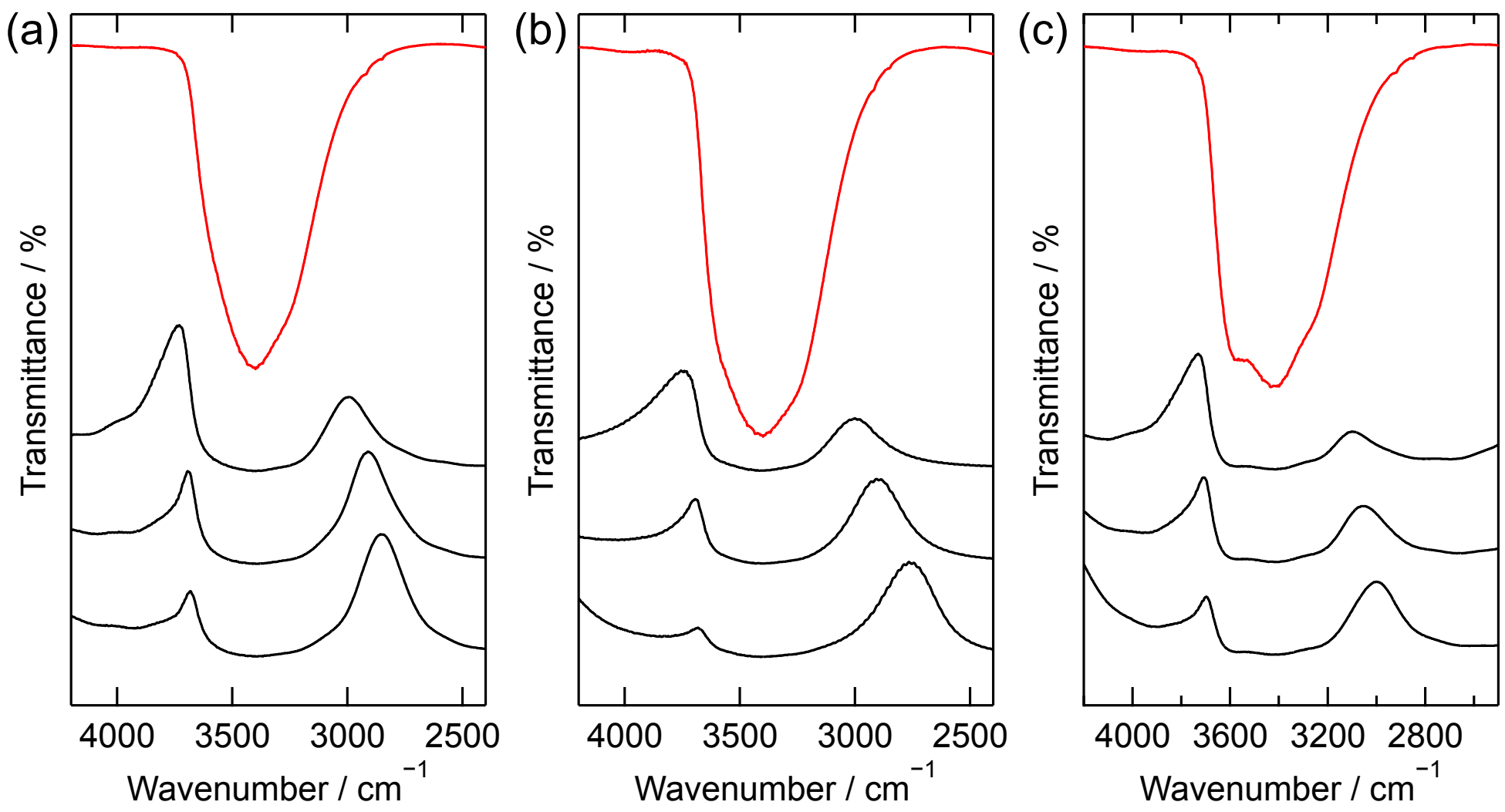

Fig. S3. IR spectra of LiTFSI solution in a non-cavity cell (red) and LiTFSI solution in a cavity cell (black). The concentration was (a) 2.1 mol L^{-1}, (b) 4.2 mol L^{-1}, and (c) 10.5 mol L^{-1}.

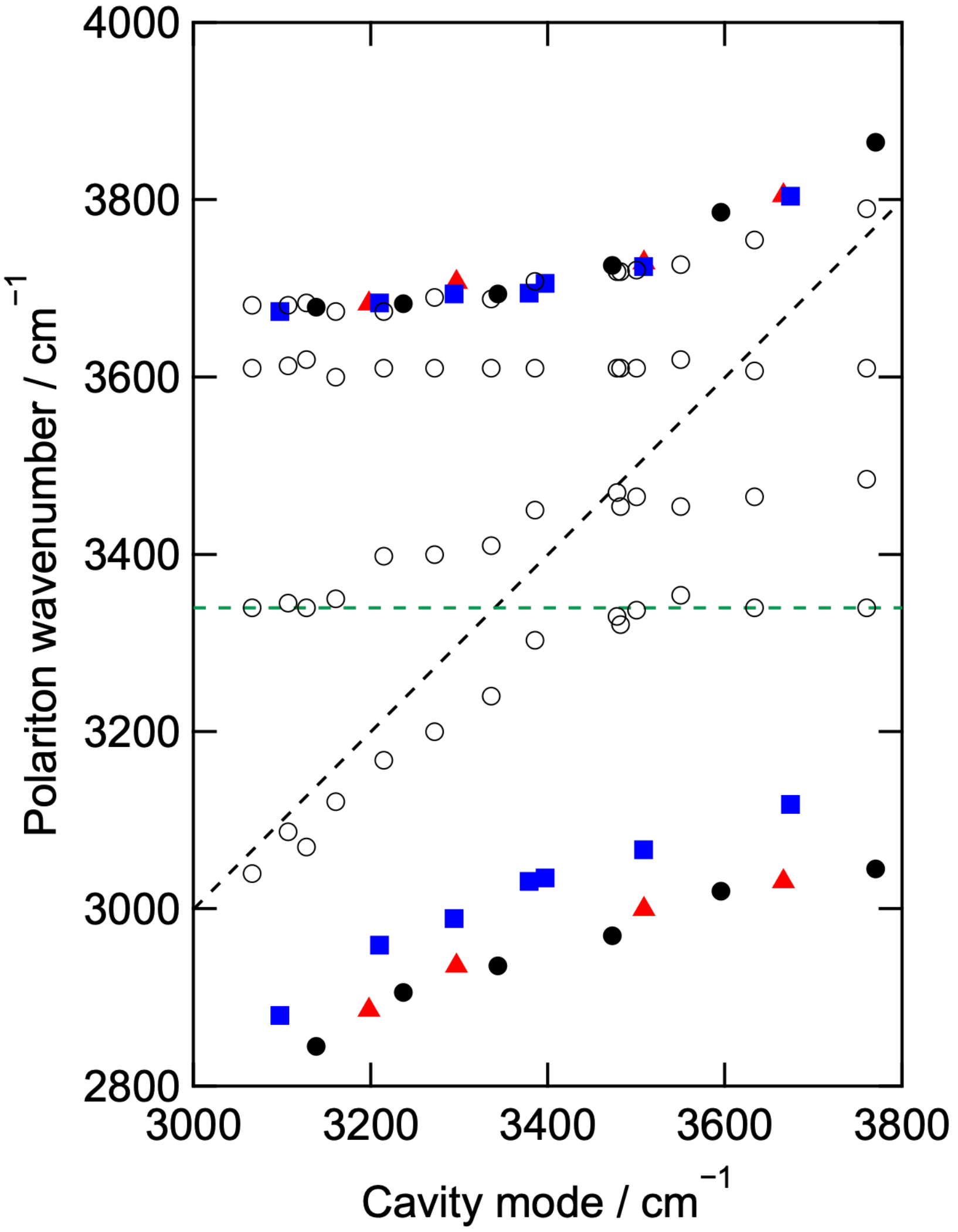

Fig. S4. Dispersion behavior of the LiTFSI aqueous electrolyte in cavity. The concertation of the electrolyte was 2.1 M (black circle), 4.2 M (red triangle), 10.5 M (blue square), and 21 M (open circle). A black dotted line represents the light line. A green dotted line represents OH stretching mode.

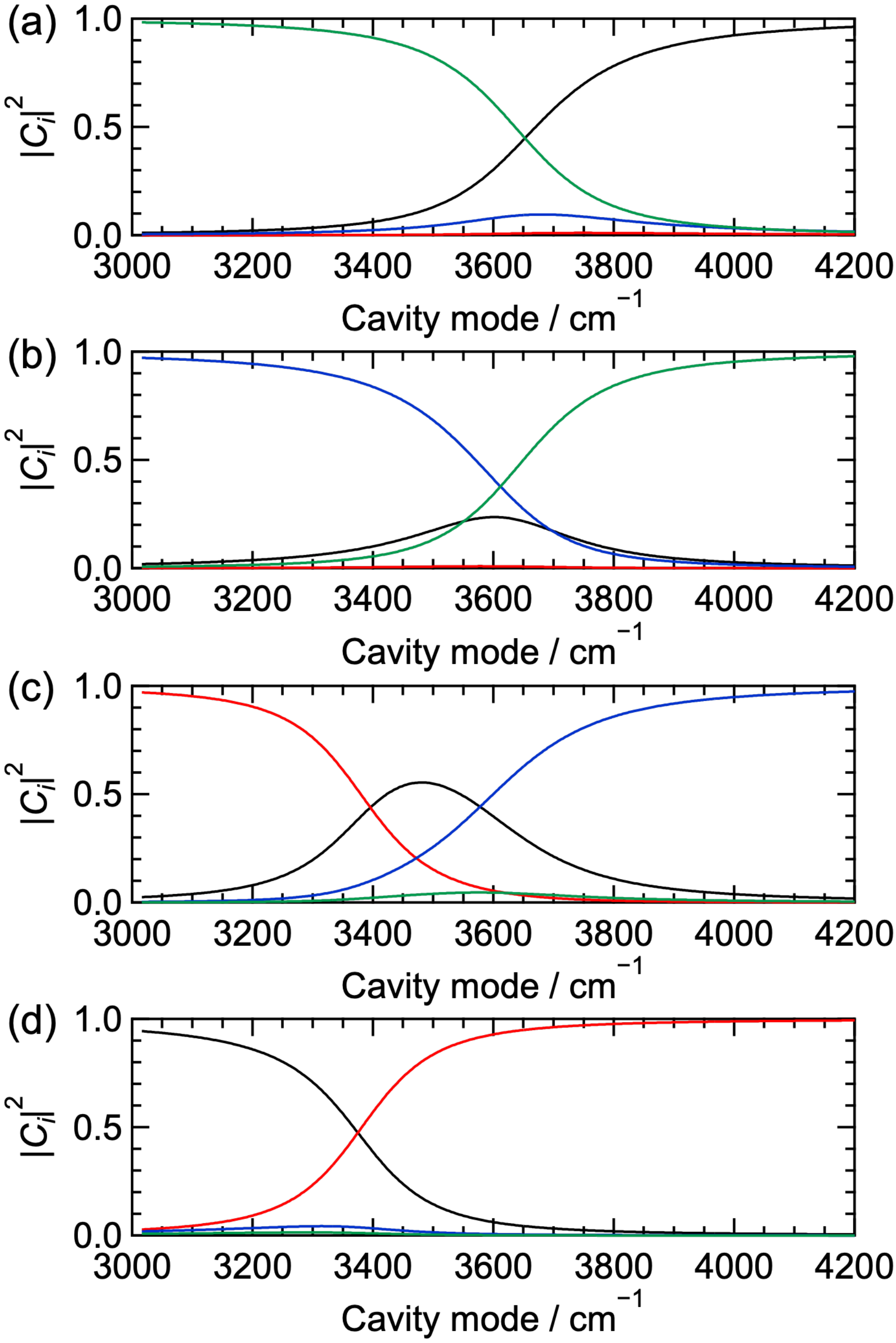

Fig. S5. Calculated photonic and vibrational fractions of the (a) P1, (b) P2, (c) P3, and (d) P4 polariton branches as a function of the bare cavity-mode frequency. The black, green, blue, and red curves represent the cavity, bulk-like water, Li^+-bound water, and $TFSI^-$-bound water fractions, respectively.

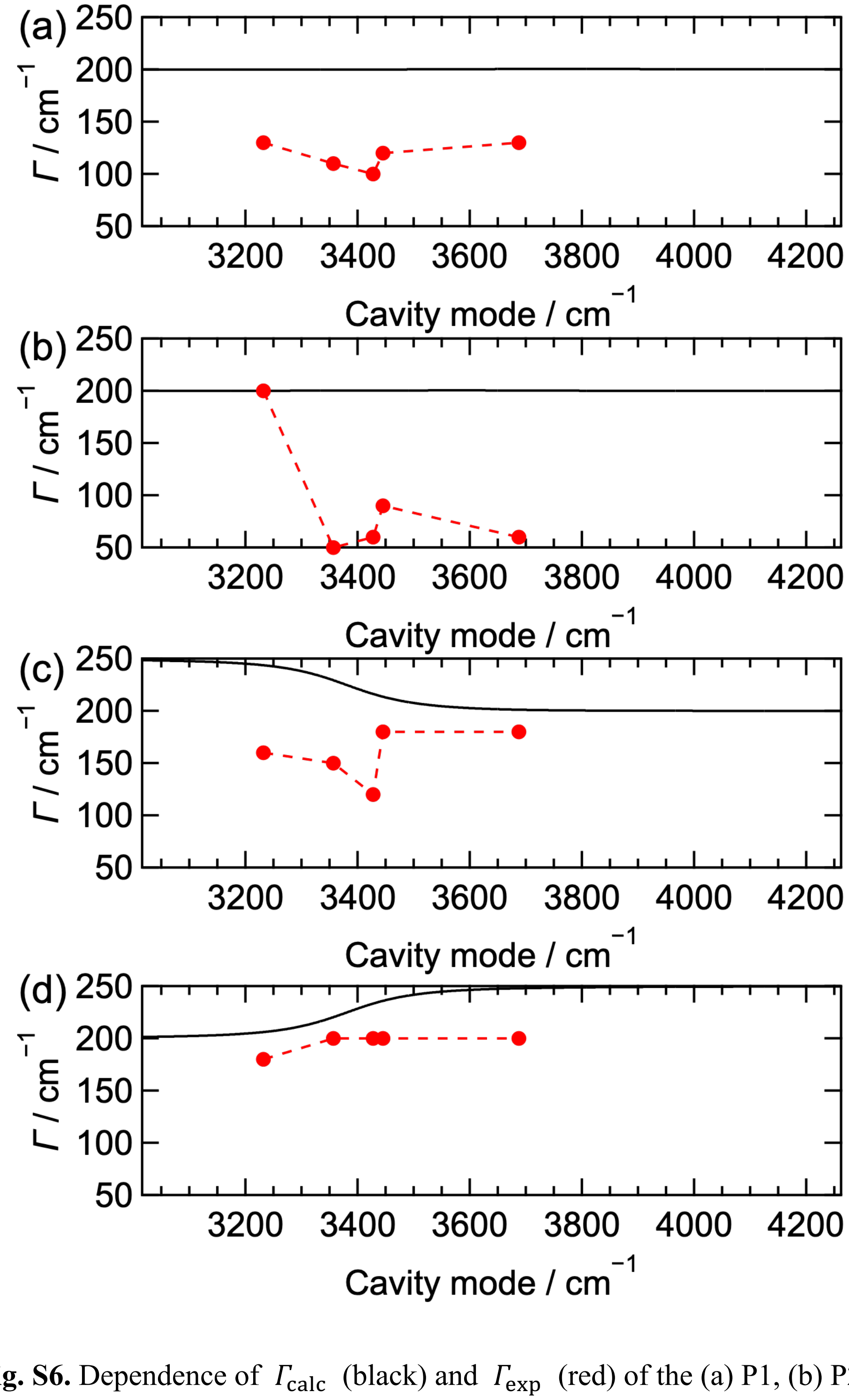

Fig. S6. Dependence of Γ_{calc} (black) and Γ_{exp} (red) of the (a) P1, (b) P2, (c) P3, and (d) P4 on cavity modes.
